# The Mitochondrial Permeability Transition Pore and Cancer: Molecular Mechanisms Involved in Cell Death

**DOI:** 10.3389/fonc.2014.00302

**Published:** 2014-11-17

**Authors:** Massimo Bonora, Paolo Pinton

**Affiliations:** ^1^Section of Pathology, Oncology and Experimental Biology, Laboratory for Technologies of Advanced Therapies (LTTA), Department of Morphology, Surgery and Experimental Medicine, University of Ferrara, Ferrara, Italy

**Keywords:** apoptosis, cancer, necrosis, permeability transition pore, ATP synthase, ROS, tumors, PTP

## Abstract

Since its discovery in the 1970s, the mitochondrial permeability transition (MPT) has been proposed to be a strategic regulator of cell death. Intense research efforts have focused on elucidating the molecular components of the MPT because this knowledge may help to better understand and treat various pathologies ranging from neurodegenerative and cardiac diseases to cancer. In the case of cancer, several studies have revealed alterations in the activity of the mitochondrial permeability transition pore (mPTP) and have determined its regulatory mechanism; these studies have also suggested that suppression of the activity of the mPTP, rather than its inactivation, commonly occurs in solid neoplasms. This review focuses on the most recent advances in understanding mPTP regulation in cancer and highlights the ability of the mPTP to impede the mechanisms of cell death.

## Introduction

The mitochondrial permeability transition (MPT) refers to an alteration in the permeability of the inner mitochondrial membrane (IMM), which was characterized for the first time in 1979 (1). The interest in this unusual mitochondrial state increased exponentially in the 1990s after the MPT was shown to be a strategic regulator of cell death (2). In the earliest study on this subject, the MPT was shown to be induced by high concentrations of mitochondrial Ca^2+^ ([Ca^2+^]_m_) and to be inhibited by Mg^2+^ and adenosine diphosphate (ADP) (1). In a later study, oxidative stress was also reported to induce the MPT (3). Thus, [Ca^2+^]_m_ and oxidative stress are currently known as the two stereotypical activators of MPT. The MPT has detrimental effects not only on mitochondria but also on the entire cell. A loss of IMM permeability leads to rapid loss of the mitochondrial membrane potential (ΔΨmt) and the consequent loss of adenosine triphosphate (ATP) as well as osmotic shock to the organelle and rupture of the outer mitochondrial membrane (OMM). The loss of ATP causes a loss of ion homeostasis and cell integrity, which finally results in necrosis (4).

Interestingly, these acute phenomena are efficiently prevented by the immunosuppressive cyclosporine A (CsA) (5, 6), indicating that the pharmacological target of CsA, the mitochondrial peptidyl-prolyl isomerase F [also known as cyclophilin D (CypD)], may play an important role in the MPT (7, 8) (see below).

It would be insufficient and inaccurate to assume that the MPT only results in necrotic cell death. The rupture of the OMM that occurs during mitochondrial swelling can lead to the release of mitochondrial proapoptotic factors, such as cytochrome C (9), apoptosis-inducing factor (AIF) (10), second mitochondria-derived activator of caspases (SMAC)/direct IAP-binding protein with low PI (DIABLO) (11), and endonuclease G (EndoG) (12), and each of these factors can induce proapoptotic activity in different ways. A role for the MPT in apoptotic cell death has been supported by several studies. For example, isolated mitochondria exposed to MPT-inducing stimuli undergo apoptotic-like morphological rearrangements when mixed with isolated nuclei (13). The MPT inhibitor bongkrekic acid (14) inhibits cell death, while the MPT inducer atractyloside (9) renders the cell more susceptible to cell death. Additionally, the proapoptotic protein B-cell lymphoma (BCL)-associated X (Bax) can induce loss of the ΔΨmt in a CsA-sensitive manner (15, 16).

Considering that ATP is a critical component in apoptosis, it is possible that the loss of mitochondrial ATP synthesis (17) as a result of the MPT would not allow MPT-driven apoptosis. This possibility is partially supported by studies showing that loss of the ΔΨmt does not occur with certain apoptotic stimuli [however, loss of the ΔΨmt is not necessarily due to an opening of the mitochondrial permeability transition pore (mPTP), and mPTP activity data based on ΔΨmt measurements must always be validated by other assays using CsA or other mPTP inhibitors]. Based on these findings, the MPT may not involve the entire mitochondrial network within the cell, and the MPT may only occur at the level of individual mitochondria (18). These isolated events could generate a localized and multi-phasic release of mitochondrial proapoptotic factors that would eventually result in apoptosis (19, 20). The cytoplasm, but not the mitochondria, may be the source of ATP during apoptosis. In this case, an apoptotic event induced by the MPT could proceed even when loss of the ΔΨmt occurs (21).

Additional information about the involvement of the mPTP in apoptotic and necrotic cell death arrive from two different studies on transgenic mice lacking the *ppif* gene, which encodes mitochondrial CypD (22). Both studies clearly indicate a strategic role for CypD in MPT-induced necrosis, as cells from *ppif*−/− mice appear to be resistant to necrosis induced by both Ca^2+^ and oxidative stress, as measured by several readouts. The relationship between the mPTP and apoptosis is less clear. Both papers clearly show that exposure of isolated mitochondria to Ca^2+^ induces cytochrome C release from *ppif*+/+ cells, while the same does not occur in *ppif*−/− cells because this release is considered one of the master events in the mitochondrial pathway of apoptosis, which suggests that the mPTP maintains a role in regulating apoptosis induction (22). Unfortunately, both papers also show no differences in apoptosis induction between the two different backgrounds (wt and ko) when induced by stimuli such as staurosporine and TNF alpha or during tBid and Bax overexpression.

The observation of a CypD-dependent cytochrome C release triggered by Ca^2+^ in a system that does not display CypD-dependent apoptosis is paradoxical. The first possible interpretation of the paradox is that mPTP opening is able to induce cytochrome C release, but the alteration of cellular homeostasis is so rapid and dramatic that necrosis overcomes apoptosis. Another possible explanation involves the dose and timing of the stimuli. For example, in the study of Nakagawa, H_2_O_2_ was used as a stimulus to induce cell death. In this case, the cells answered by engaging necrosis but not apoptosis; however, it has been extensively reported that hydrogen peroxide treatment is also able to induce apoptosis [for instance, see in Ref. (23–26)]. Finally, it should be considered that CypD is not a structural component of mPTP, but is one of its regulators (maybe the most investigated of all), and its role in the complex is to regulate the threshold for Ca^2+^; however, mPTP opening is still achievable. Because the mPTP opening mechanism in response to stimuli such as Bax and Bid is unclear, these proteins may not require Ca^2+^ to trigger the MPT and thus would not require CypD.

Overall, studies on *ppif* ko mice clearly show that Ca^2+^ is a trigger for MPT-induced necrosis, but these studies do not exclude the possibility that MPT is an apoptotic inducer that responds to other types of triggers.

In conclusion, although the specific type of cell death caused by the MPT has not been confirmed, the mere existence of the MPT indicates that mitochondria are master regulators of danger signals and are capable of transducing life or death signals due to their interconnection with Ca^2+^ signaling (27–31).

## Background on mPTP Structure

The mPTP is the putative pore responsible for the MPT, an event in which the mitochondrial inner membrane, which is highly impermeable, becomes extremely permeable. The initial model of the mPTP proposed that the voltage-dependent anion channel (VDAC) and the adenosine nucleotide transporter (ANT) were located on the OMM and IMM, respectively and that they were core components of the pore. These proteins are surrounded by a series of regulators, including kinases such as hexokinase II (HKII), creatine kinase (CK), and glycogen synthase kinase 3 β (GSK3β) (32); the translocator protein (TSPO); CypD; and members of the Bcl-2 family (22, 33). In particular, the proapoptotic members Bax and Bcl-2 homologous antagonist killer (Bak) have a dramatic positive effect on mPTP opening, as confirmed in *bax* and *bak* knockout models (34). The role of these proteins in the regulation of MPT is likely to depend on their ability to permeate the OMM, which was partially confirmed in an older study illustrating that the removal of Bax and Bak led to impaired mitochondrial Ca^2+^ uptake (35).

The results from VDAC and ANT knockout studies in animal models, however, have demonstrated that these elements are not pore-forming components; thus, they have been categorized in the broad group of activity regulators.

The observation that inorganic phosphate sensitizes the mPTP suggests that a Pi-binding protein could be associated with the pore. For a long time, it was thought that this component was an inorganic phosphate transporter (PiC) based on the observations that: (i) a non-specific pore is generated in liposomes by reconstituting the PiC (36), (ii) the PiC interacts with mitochondrial CypD and ANT (37), (iii) this interaction is strengthened by MPT-inducing agents, whereas MPT-blocking compounds diminish the interaction, and (iv) PiC overexpression induces mitochondrial dysfunction and apoptosis (38). These results identified PiC as a strong candidate for the core-forming element of the mPTP.

Recent knockdown/knockout experiments performed both *in vitro* (39) and *in vivo* (40, 41) have verified that PiC cannot be the core component; rather, it serves as an additional regulator.

The sensitivity of the mPTP to inorganic phosphate also drew our attention to respiratory complex I, NADH:ubiquinone oxidoreductase (hereafter referred to as Complex I). It has been observed that the Complex I inhibitor rotenone is also an inhibitor of the mPTP, and its effect is dependent on the Pi level (42). Inhibition of the mPTP by rotenone is apparently linked to the activity of Complex I rather than to the production of reactive oxygen species (ROS) or depletion of pyridine nucleotides (43). Further, the relationship between Complex I activity and mPTP inhibition appears to be correlated with structural rearrangements of Complex I (44). This finding led Fontaine’s group to propose that respiratory Complex I could act as a negative regulator of mPTP via a direct interaction, which would depend on the activity of Complex I and the availability of substrates (42).

Overall, these findings have provided several hypotheses regarding the regulation of the mPTP; however, a feasible structural model of the mPTP is still lacking.

The results from a series of studies suggest that mitochondrial F_1_/F_O_ ATP synthase (hereafter referred to as ATP synthase) may be an important component of the mPTP (45–49). Our group was among the first to demonstrate that the C subunit of mitochondrial ATPase is a fundamental regulator of mPTP activity (50, 51). Inhibiting C subunit expression completely blocks the induction of the MPT by Ca^2+^ and oxidants, and overexpressing the C subunit dramatically promotes MPT induction.

Initially, we speculated that the C-ring forms the core of the mPTP. This speculation was supported by a subsequent study demonstrating that currents generated by isolated C subunits on artificial bilayers and in isolated mitochondria are sensitive to mPTP regulators (52). Despite these findings, no studies have yet shown that C-rings can exist outside of ATP synthase *in vivo* or that they can form “free C-rings” that are capable of generating currents.

**Figure 1:**
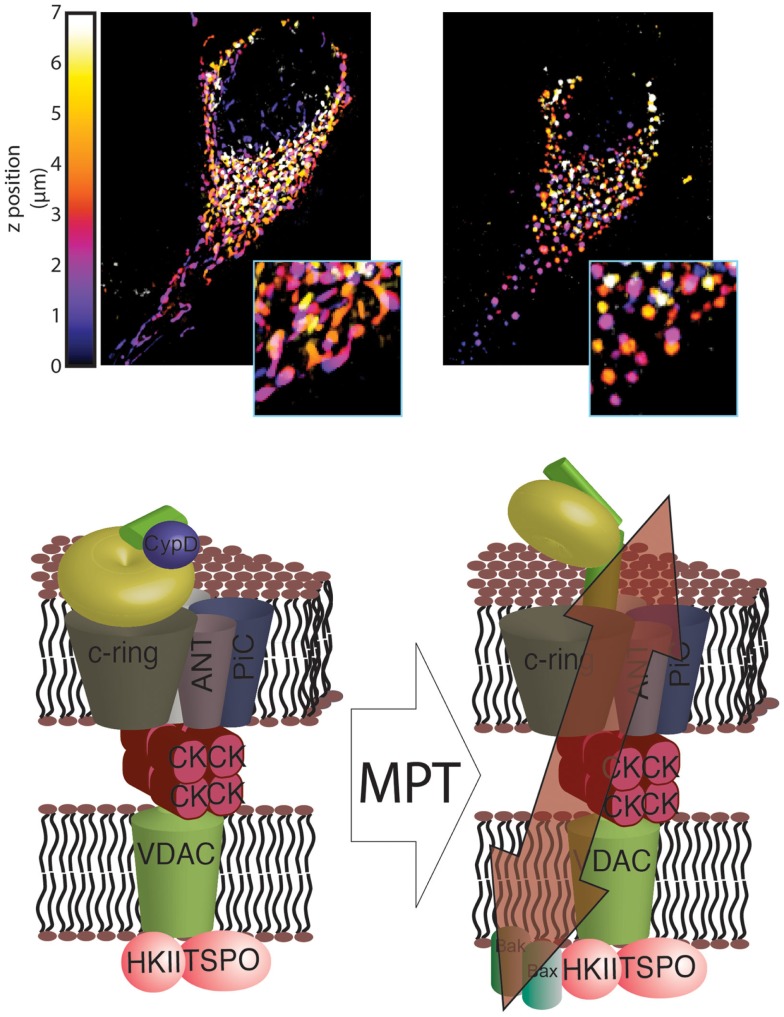
**Novel structure of the mPTP**. In healthy mitochondria, which are characterized by a dense and interconnected network (top left), the F_1_/F_O_ ATP synthase is organized into super-structures with the known mPTP regulators ANT and PiC. These regulators, in turn, are able to interact with other mPTP regulators, including HKII, VDAC, TSPO and CK (bottom left). Induction of MPT results in a reorganization of the F_1_/F_O_ ATP synthase structure that allows its C-ring to generate a non-specific current, which leads to mitochondrial network fragmentation and generation of circular mitochondria (mitochondrial swelling, top right). These events, which are usually associated with HKII detachment from the outer mitochondrial membrane (OMM), are facilitated by interaction with GSK3 and by insertion of Bax and Bak into the OMM (bottom right).

Finally, a recent study by Alavian et al. (53) demonstrated how the C-ring can generate a non-specific current attributable to the mPTP. Specifically, these authors found that isolated F_1_/F_O_ ATP synthase monomers reconstituted on vesicles can generate currents when bound to CypD and exposed to Ca^2+^. Additionally, sub-mitochondrial vesicles enriched in ATP synthase can also generate a current that is sensitive to Ca^2+^ and CsA, and removing the F_1_ subunit can abolish this sensitivity. Furthermore, Ca^2+^-induced mitochondrial swelling can cause the F_1_ subunit to partially detach from the F_O_ subunit, and CsA can reverse this partial detachment. Finally, Alavian et al. further speculated that the C-ring rearranges during the induction of the MPT such that its diameter is increased, forming a non-specific channel (Figure 1).

These findings emphasize the role that ATP synthase, and especially its C-ring, plays in mPTP formation but do not provide a clear structural model for the mPTP. First, the precise conformational rearrangements that promote the generation of a non-specific current must be determined. Until now, only a rearrangement of the C-ring, with the potential involvement of the β subunit, has been proposed. Second, the mechanism underlying this conformational change in ATP synthase must be elucidated, and any other subunits that play a role in this rearrangement must be identified. Finally, the roles played by known components of the mPTP must be determined. For example, the mechanism by which targeting of the mPTP regulators affects the sensitivity of pore opening remains unknown.

Although more studies are required, our understanding of the mPTP has improved since it was first identified.

## Molecular Aspects of the mPTP in Tumor Development

Evasion of apoptosis is recognized as one of the hallmarks of cancer and is required for the development and progression of the pathology (54). The mPTP can initiate cell death; thus, it may play a role in cancer.

When considering the potential role of the mPTP in cancer, investigators often question whether changes in mPTP activity promote tumor initiation and progression. Several studies on the mPTP have been conducted in cancer cell lines derived from cervical cancer (50), colon cancer, osteosarcomas (55), leukemia (56), and many other cancers, and these studies have shown that loss of the mPTP itself is not sufficient for cancer development. Despite this observation, several inducers, and other mPTP regulatory events are inefficient in tumor cells. We will discuss a few of these cases in this section.

### Alterations in MPT inducers

The best-known MPT inducer is intra-mitochondrial calcium. Stimuli that elevate the mitochondrial Ca^2+^ concentration for a sustained period of time induce the MPT and cell death (57, 58). Ca^2+^ is provided to mitochondria by the endoplasmic reticulum through specialized domains in which the two organelles make contact via mitochondria-associated membranes (MAMs) (27, 28, 59).

**Figure 2:**
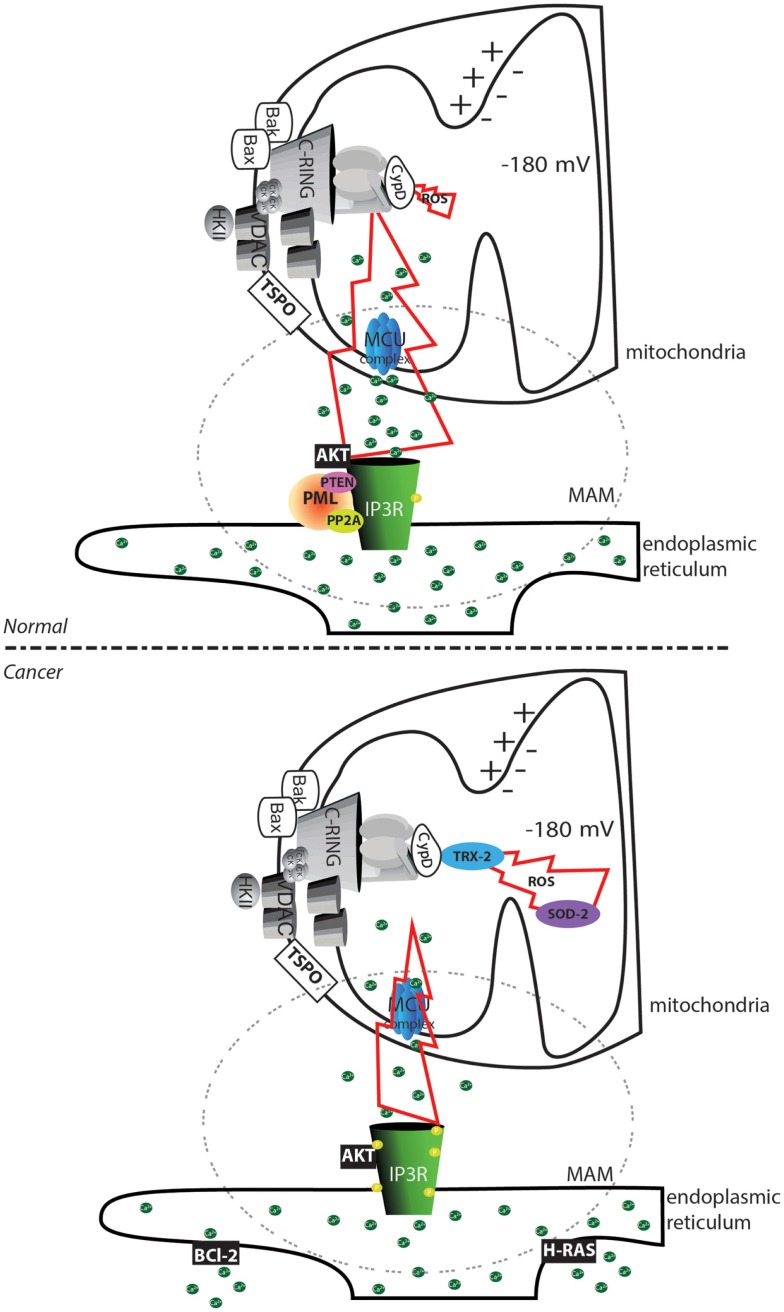
**Altered mPTP activation in cancer cells**. In normal cells, the transfer of substances between the endoplasmic reticulum and mitochondria is finely regulated by the phosphorylation status of the IP3Rs. Phosphorylation can be mediated by the AKT kinase, and proteins are dephosphorylated by the PP2A phosphatase. This process is regulated by the oncosuppressors PML and PTEN. In cancer cells, the loss of PML or PTEN allows an increase in the phosphorylation of IP3R that inhibits Ca^2+^ transfer between the endoplasmic reticulum and the mitochondria. Further accumulation of the oncogenic proteins Bcl-2 and RAS in the endoplasmic reticulum allows depletion of the Ca^2+^ store. The reduced amount of transferable Ca^2+^ prevents the occurrence of the MPT, despite the increased ROS levels often observed in cancer cells. Further, the increase in the levels of the ROS detoxifying enzymes superoxide dismutase-2 (SOD-2) and mitochondrial thioredoxin reductase (TRX-2) reduces the toxicity of oxidative stress. The region of contact between the mitochondria and ER, corresponding to the MAMs structure, is marked with a dashed circle. The yellow circle represents phosphorylated residues. Green circles represent Ca^2+^ ions.

Our group and others have demonstrated that cancer cells evade this type of signaling via several mechanisms. The oncosuppressors promyelocytic leukemia protein (PML) (60) and phosphatase and tensin homolog (PTEN) (61), in cooperation with protein phosphatase 2A (PP2A), sustain the Ca^2+^ transfer between the ER and mitochondria through the mitochondrial Ca^2+^ uniporter (MCU) complex (62) by regulating the phosphorylation state of the channel responsible for the Ca^2+^ release: the inositol 3 phosphate receptor (IP3R) (63, 64). Loss of these regulators inevitably reduces the probability of properly initiating the MPT (31). In contrast, Bcl-2 reduces the Ca^2+^ content in the ER (65), and the mitogenic kinase AKT strongly inhibits the IP3R; both of these factors favor the inhibition of cytotoxic Ca^2+^ signals (Figure 2).

It appears that tumor progression is sustained by the accumulation of a series of changes in the Ca^2+^ regulatory machinery that decrease the cytotoxic Ca^2+^ signal. In support of this idea, Rimessi et al. showed that tumor transformation resulting from the oncogenic activation of the Harvey rat sarcoma viral oncogene homolog (H-RAS) (66) is accompanied by a progressive reduction in the amount of intracellular Ca^2+^ that is transferable to mitochondria. Furthermore, these authors also demonstrated that a controlled increase in the extracellular Ca^2+^ level causes an increase in the intracellular Ca^2+^ level, which impedes H-RAS-induced transformation (67).

The second most important inducers, which were previously mentioned, are ROS. The current findings on ROS-induced cell death and cancer inevitably lead to the following paradox: many cancer cells have higher basal ROS levels compared with normal cells, but cancer cells are highly resistant to cell death. One explanation for this paradox is that ROS also promote several hallmarks of cancer, such as proliferation, invasion, and metastasis [for more details, see in Ref. (68) and (69)]. Another explanation is that several cancer cell types have higher levels of antioxidants, which could inhibit ROS toxicity; specifically, increased levels of superoxide dismutase (SOD-2) and thioredoxin reductase 2 (TRX-2) were observed in mitochondria from cancer tissue samples (70–72).

Furthermore, an elevated ROS level may reduce the mPTP threshold for Ca^2+^. Therefore, the decrease in the Ca^2+^ level that occurs during cell transformation may allow the ROS level to increase without alerting the cellular regulatory mechanisms. Evading these regulatory mechanisms would then allow the tumor cell to undergo the cancer-promoting changes induced by a high ROS level.

### Levels of mPTP components

Another paradox concerning the involvement of the mPTP in cancer is related to the expression levels of its components. Studies in cancer cell lines and tumor models have shown that different mPTP proteins are overexpressed (73, 74). For example, the expression levels of VDAC isoforms are significantly higher in malignant tumor cells (75), and there is a positive correlation between tumorigenesis and the expression level of TSPO, the ligand of which can induce apoptosis in cancer cells (76, 77). Furthermore, the adenine nucleotide translocator ANT-2 is upregulated in renal tumors and transformed hepatocytes (78). This upregulation, however, may be explained by regulation of the metabolic status and may not be dependent on the MPT. Analogous to ANT-2 is HKII, the catalyst of the first step of glycolysis. This enzyme is located on the cytoplasmic side of the mitochondrial outer membrane, where it interacts with other mPTP regulators, and removing this enzyme from this location causes the mPTP to open (79, 80). The expression level of HKII is higher in several tumors (81–84). However, because of its role in glycolysis, the upregulation of HKII expression may be linked to the Warburg effect, which is generally observed in tumors and provides a metabolic advantage rather than specific resistance to apoptosis.

These findings suggest that the expression levels of the mPTP components are generally higher in tumors, and this increase is likely to enhance the probability of mPTP formation and cell death, in contrast to the reduced sensitivity to apoptosis that is typical of tumors. The solution to this enigma may be found within the Bcl-2 family of proteins.

**Figure 3:**
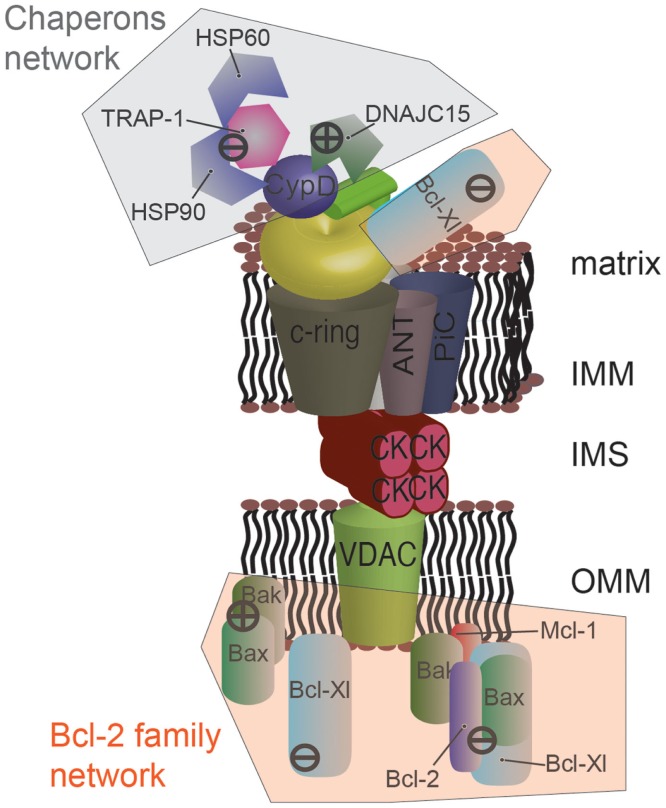
**Protein networks associated with the mPTP in cancer**. Several members of the Bcl-2 family can negatively modulate mPTP opening via direct interaction. Specially, the proapoptotic proteins Bax and Bak are sequestered by the anti-apoptotic proteins Bcl-2, Mcl-1, and Bcl-Xl. Bcl-Xl displays a higher degree of regulation, and its direct interaction with the β subunit of F_1_/F_O_ ATP synthase is also considered to inhibit mPTP opening. A network of chaperones has also been uncovered at the mitochondrial matrix side. The fundamental mPTP regulator CypD, a chaperone itself, is able to interact with HSP90, TRAP-1, and HSP60. This complex appears to inhibit CypD, promoting the closed state of the pore. A similar situation was observed for DNAJC15, which interacts with CypD and inhibits the MPT. The question of whether these chaperones are organized into a unique supramolecular complex remains to be investigated.

The Bcl-2 family is a well-studied group of proteins involved in the regulation of apoptosis, necrosis, and autophagy (85, 86). Within this family, several anti-apoptotic members, such as B-cell lymphoma-extra large (Bcl-Xl) and myeloid cell leukemia 1 (Mcl-1), are overexpressed in cancer (87). Bcl-Xl has been shown to negatively regulate mPTP opening by directly interacting with VDAC (88) at the cytoplasmic side. More recently, the group of Jonas identified an unexpected mitochondrial-matrix-localized form of Bcl-Xl that is able to interact with the β subunit of ATP synthase to promote its synthetase activity and to inhibit mPTP opening in parallel [Figure 3; (89)].

Although the precise mechanism has not been elucidated, it has been shown that Mcl-1 plays a role in inhibiting the MPT (90). Furthermore, it has been proposed that the anti-apoptotic members Bcl-2, Bcl-Xl, and Mcl-1 interfere with the proapoptotic interactions induced by Bax and Bak (Figure 3). Specifically, it has been suggested that Bax and Bak positively regulate the opening of the mPTP (15, 34, 91). Mutations in the Bax protein have been identified in gastric and colon cancer (92, 93), and its degradation is accelerated in prostate cancer (94). Missense mutations in Bak have been identified in late-stage gastrointestinal cancer (95). Unlike normal cells, pancreatic cancer cells are unable to properly increase Bak expression in response to stimuli, which suggests that the Bax-dependent apoptotic pathway is inhibited (96).

Therefore, it appears that an increase in the mPTP components in cancer is counteracted by an increase in negative regulators belonging to the Bcl-2 family or by inactivation of positive regulators belonging to the Bcl-2 family, which may be another strategy for evading MPT-induced cell death.

### Chaperone network around the mPTP

The gold standard in MPT studies is the use of CsA as a potent inhibitor of the mPTP. This immunosuppressant is a target of CypD, a mitochondrial chaperone that is required for proper opening of the mPTP and that confers Ca^2+^ sensitivity to the complex. Although its mechanism has not yet been elucidated, it has been suggested that CypD promotes the rearrangement of the mPTP subunits to allow for channel formation because it is a chaperone protein. Furthermore, other mitochondrial chaperones may also be involved in a similar mechanism.

Accordingly, heat shock protein 90 (HSP90) was found in a complex with TNF receptor-associated protein-1 (TRAP-1) and CypD (97). This complex appears only in tumors and inhibits apoptosis. Furthermore, the use of mitochondria-targeted ATPase antagonists is sufficient to block the protection provided by HSP90 and to restore the susceptibility of the cell to apoptosis. Although mPTP activity was not measured, it was speculated that this complex is part of a novel cytoprotective pathway that involves the MPT and is required for tumor growth *in vivo* (Figure 3). Another study detected the mitochondrial chaperone heat shock protein 60 (HSP60) in this same complex (98). This finding received significant interest because HSP60 is often overexpressed in tumors (99).

Recently, DnaJ homolog, subfamily C, member 15 (DnaJC15) has been implicated in control of the MPT. J proteins are chaperones that cooperate with heat shock protein 70 (HSP70) to direct proteins to specific intracellular locations, such as mitochondria, and DnaJC15 is part of the mitochondrial protein transport machinery. Ovarian and breast cancer cells from patients exposed to chemotherapy displayed reduced levels of DnaJC15 and showed enhanced resistance to the therapy. This resistance was attributed to methylation of CpG islands in the gene encoding DnaJC15. DnaJC15 has not been found to have a role in cell death or proliferation.

Sinah and D’Silva (100) demonstrated that DnaJC15 regulates mPTP activity by interacting with CypD (Figure 3). These authors observed that DnaJC15 overexpression induced spontaneous MPT, while its downregulation (as observed in cancer) reduced the sensitivity of mPTP opening when stimulated with cisplatin.

Thus, the notion is emerging that a complex chaperone network develops in the mitochondria of tumor cells that inhibits mPTP opening. The existence of this network would at least partially account for the different chaperone levels that are observed in several types of cancers (101) and would provide a new panel of pharmacological tools that could selectively target tumors and reduce the toxicity of the current anticancer strategies (102).

### Afferent signaling to the mPTP

A group of well-known oncogenes and oncosuppressors are able to modulate Ca^2+^ homeostasis to regulate induction of the MPT. Interestingly, some of these factors are able to localize to MAMs, potentially to increase the selectivity and efficiency of their regulatory effect. This is the case for AKT (103) and two oncosuppressors, PML (63), and PTEN (64). At these sites, AKT, PML, and PTEN appear to exist in a complex that interacts with and determines the phosphorylation status of the IP3R, which affects its channel activity (Figure 2). This model has received a significant amount of interest because AKT and PTEN are both members (with opposite functions) of the PI3K pathway, which is one the most well-studied survival signaling pathways and plays a critical role in resistance to anticancer therapies (104–106).

**Figure 4:**
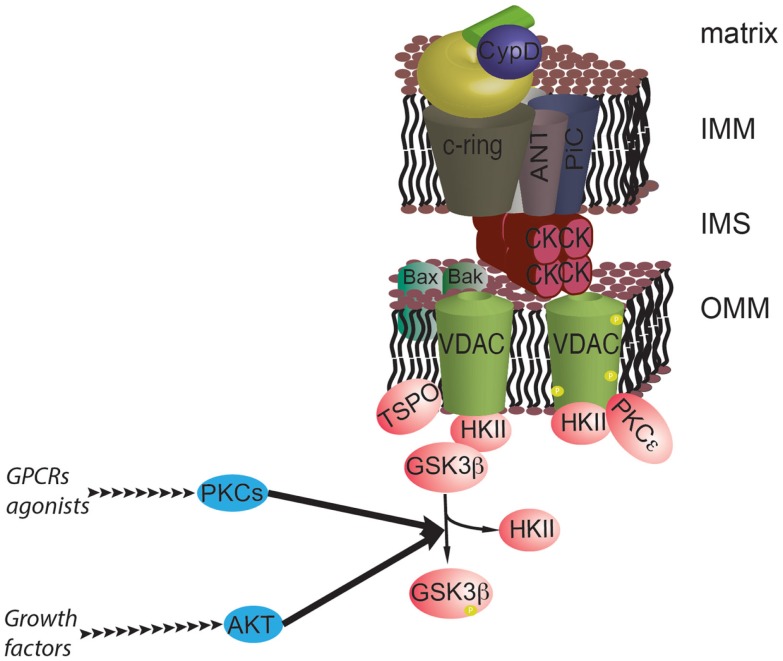
**The prosurvival pathway converges on the mPTP**. GSK3β is a negative regulator of the mPTP, and its activity is inhibited by phosphorylation by AKT and PKCs. GSK3β functions as a hub for different survival signals at the interface with mPTP. In addition, PKCε can stabilize the interaction with HKII by phosphorylating VDAC.

AKT may affect mPTP activity through another mechanism that is not directly related to Ca^2+^ signaling. AKT can induce the phosphorylation of GSK3β, which results in its inactivation. GSK3β kinase has a significant stimulatory effect on the mPTP. Its inactivation by AKT strengthens the association between HKII and VDAC, causing inhibition of mPTP function and an increase in cell survival (107). Alterations in GSK3β have been found in several cancer types [for details, see in Ref. (108)]; furthermore, GSK3β is of significant interest because it could act as a convergence factor that allows survival signaling pathways to interact with the mPTP. In addition to the growth signals stimulated by AKT, the wingless integrated human homolog (WNT), protein kinase C (PKC), and extracellular signal-regulated kinase (ERK) pathways can also regulate GSK3β phosphorylation, which potentially connects the MPT to signals stimulated by growth factors, ligands of G protein-coupled receptors, and the extracellular matrix [Figure 4; (109, 110)].

To further emphasize the potential role of GSK3β in regulating the mPTP in cancers, the activation of GSK3β causes the release of HKII from the OMM. The use of synthetic inhibitors showed that this event can induce cell death in cancer cell lines (79).

The epsilon isoform of PKC (PKCε) has been proposed to have a mechanism of action that is independent of GSK3β. First, PKCε was proposed to form a complex with VDAC1–HKII–ANT, thereby inducing VDAC1 phosphorylation and the consequent inhibition of mPTP activity (Figure 4). However, PKCε was also shown to reduce the Ca^2+^ content in the sarcoplasmic reticulum during preconditioning of cardiac tissue. This is likely to reduce the probability of mPTP induction during ischemia/reperfusion, conferring a mechanism for preconditioning-mediated cardioprotection (111). The question of whether this event could be relevant in the tumor environment has yet to be addressed.

mTOR, a negative regulator of autophagy, functions downstream of the AKT pathway. Targeting mTOR inhibits the mPTP to a similar extent as activating AKT and inactivating GSK3β, and inhibition of mTOR enhances the binding of HKII to VDAC (112).

Thus, kinase cascades that are significantly activated in cancers can maintain HKII bound to the mitochondrial surface via inhibiting GSK3, thus keeping the mPTP closed. These events may contribute to the anti-apoptotic phenotype of tumor cells and to the development of resistance to therapies. In contrast, strategies directed at activating GSK3 may increase the sensitivity of mPTP opening and increase the probability of tumor regression (113).

### mPTP and the Warburg effect

Mitochondrial permeability transition-driven cell death is closely associated with the hypoxia-induced ischemia that occurs in normal tissue as well as the consequent reperfusion injury. The current model proposes that during ischemia, the accumulation of lactate resulting from oxygen deprivation leads to a decrease in the pH and activation of the Na^+^/H^+^ exchanger, which is required to buffer the cytoplasmic pH. Accumulation of Na^+^ is then prevented via the action of the Na^+^/Ca^2+^ exchanger, which increases the cytosolic Ca^2+^ concentration ([Ca^2+^]_c_). The accumulation of cytoplasmic Ca^2+^ is further increased by a reduction in the activity of the Ca^2+^ pumps located on the sarcoplasmic reticulum and sarcolemma because of the loss of ATP. Furthermore, the sustained loss of ATP leads to temporary accumulation of ADP, which is converted to adenine monophosphate (AMP) and adenosine and results in the accumulation of Pi. It has been shown that Pi induces the MPT. During ischemia, cells accumulate MPT inducers (Ca^2+^, Pi) and lose one of the most important MPT inhibitors, ADP (114). The most detrimental effects, however, are not observed until reperfusion, when a significant amount of ROS is produced that definitively and dramatically induces the MPT and necrosis. Based on these findings, induction of the MPT has been monitored during myocardial (115) and brain reperfusion injury (116).

The unregulated growth of a solid tumor mass generates a hypoxic environment. This condition is not irreversible; rather, it is intermittent and characterized by cycles of hypoxia and re-oxygenation (117). This condition should induce the same mechanism of mPTP opening that has been observed in the myocardium and brain and should thus reduce tumor size. These events, however, have not been observed in actual solid cancers, which suggests that a different protection mechanism is involved in these tumors.

**Figure 5:**
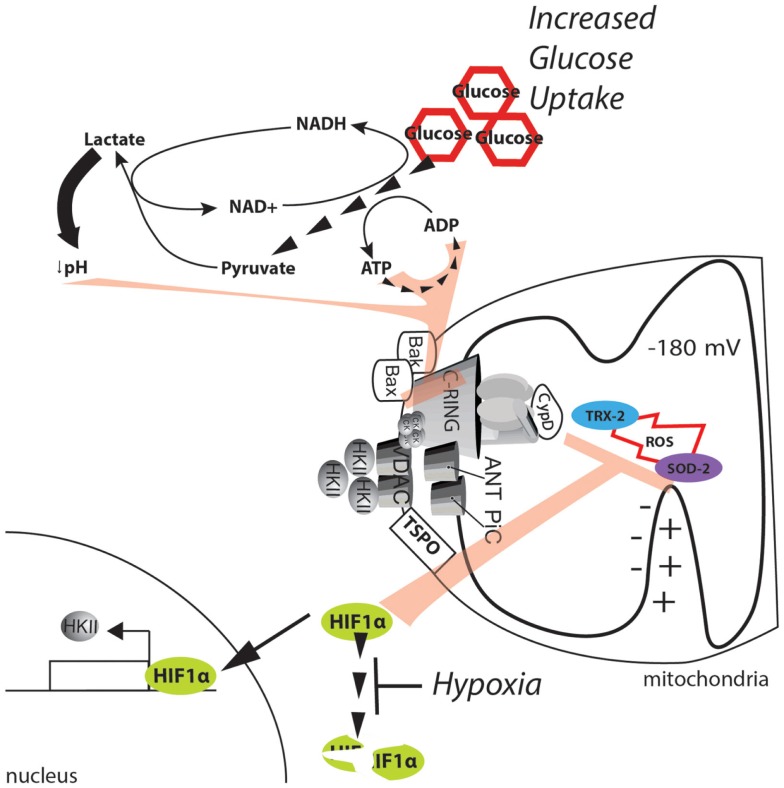
**Metabolic alterations in the cancer environment affect mPTP activity**. The dramatic increase in glucose uptake due to the Warburg effect boosts glycolysis, sustaining the ATP/ADP exchange due to metabolic reactions. Further, the high levels of pyruvate promote lactate production and cytosol acidification. ATP, ADP, and a low pH can synergistically exert strong inhibitory effects on the MPT. In addition, the hypoxic environment generated in solid tumors stabilizes the HIF1α transcription factor and allows the expression of genes involved in buffering of ROS. Further, HKII is also upregulated, and its binding to the mPTP is enhanced, thus stabilizing the closed state of the pore.

One potential protection mechanism is related to the Warburg effect, which is the dramatic increase in glucose uptake and lactate fermentation observed in tumor masses (118, 119). Solid tumors display an extraordinary capacity for glucose uptake, which is a property that is exploited for the diagnosis of solid tumors. Glucose is converted to pyruvate, which is further reduced to lactate to complete the lactate fermentation process. These reactions occur even in the presence of functional mitochondria; thus, their purpose and significance remains controversial (120, 121). Nonetheless, the findings reported by Warburg have been confirmed by other groups and result in two main consequences for the MPT. First, the increase in glucose uptake allows for the continued synthesis of ATP through glycolysis, which impedes the depletion of adenine nucleotides and Pi accumulation that were described in the previous model. Second, the accumulation of lactate lowers the pH, thus inhibiting the mPTP. This finding suggests that the Warburg effect alone may result in mPTP inhibition (Figure 5).

A more direct potential mechanism involves hypoxia-inducible factor 1α (HIF1α). HIF1α is a heterodimeric protein that acts as an oxygen-level sensor because its degradation is prevented by ROS accumulation. Additionally, HIF1α activates the transcription of genes that favor glycolysis and lactate fermentation while suppressing mitochondrial respiration [for further review, see in Ref. (122)]. The reduction in the mitochondrial potential resulting from a reduction in mitochondrial respiration inhibits Ca^2+^ uptake and the significant increase in ROS. Both of these events activate the mPTP (see above); however, because of its ability to stabilize HKII, the effect of HIF1α on the MPT is even more direct (123). Gwak and colleagues demonstrated that hypoxia promotes the expression of HKII, which inhibits the mPTP through HIF1α (Figure 5). Consistent with this finding, the selective inhibition of HKII induces apoptotic cell death, which is analogous to the results observed when HKII is induced to detach from mitochondria (79). Unfortunately, the authors did not directly demonstrate the involvement of the mPTP in this mechanism.

Older biochemical data indicate that when HKII is bound to the mPTP, glucose can induce mPTP inhibition; on the other hand, glucose-6-phosphate, which is the product of HKII, induces detachment of HKII from mitochondria (124). Because HIF1α can promote both glucose uptake (by elevating the levels of the glucose transporter GLUT1) and its conversion through glycolytic flux, it can be speculated that elevation of HIF1α in cancer will further inhibit mPTP activity by increasing the levels of glucose (to be considered as an inhibitor of mPTP) but can also impede the accumulation of glucose 6-phosphate (which can serve as an mPTP inducer) through the regulation of glycolytic enzymes and inhibition of pyruvate dehydrogenase (PDH).

Finally, the reduced oxygen levels in the tumor environment should decrease the activity of the respiratory chain and Complex I. We previously mentioned that Complex I inhibition can result in mPTP inhibition; thus, it can be postulated that hypoxia may impair mPTP activity via its effects on Complex I. However, it should be noted that the role of Complex I activity in cancer is controversial. Mutations in mitochondrial DNA that impair Complex I function reveal that the activity of Complex I is essential for stabilizing HIF1α and for generating the Warburg phenotype (125). Concurrently, another group observed that overexpression of Ndi1 (a subunit of yeast Complex I with dehydrogenase activity) favors mitochondrial metabolism in breast cancer cells and reduces breast cancer tumor formation and metastasis (126). Both studies accounted for the effect on the NADH/NAD+ ratio: the first study indicated that a decrease in the NADH/NAD+ ratio could lead to a decrease in α ketoglutarate (required for the stabilization of HIF1α), and the second study showed that NAD+ could regulate mTORC1 and autophagy, leading to inhibition of tumor growth. It should be noted that in several types of cancer, mutations in mitochondrial DNA have been reported (127–130), and the ability of these mutations to increase ROS seems to confer an advantage to tumors, especially during metastasis formation (131).

Overall, the findings from previous studies suggest that inhibition of the mPTP plays a role in the resistance of solid tumors to hypoxic conditions.

## Concluding Remarks

Because defects in the cell death pathway are considered a hallmark of tumor initiation and progression, the MPT is a potential target for rescuing oncogenic defects. In-depth studies on mPTP activity have been conducted to identify the factors that inhibit the activation of cell death pathways.

Functional mPTPs can be found in cancer cell lines, indicating that mPTPs are not completely inhibited in cancer. Furthermore, several structural elements of the pore are overexpressed and not downregulated in these cell lines. These findings are most likely the result of the paradoxical nature of the mPTP. More recent studies have proposed the existence of an “evolved aberrant conformation” of extremely efficient components that are indispensable for mitochondrial physiology (such as ion and solute transporters as well as chaperones).

Nevertheless, several mPTP regulatory mechanisms are impaired in tumors compared with the surrounding tissues. These alterations can be categorized as: (i) alterations in the concentrations of mPTP inducers (i.e., Ca^2+^), (ii) promotion or inhibition of regulators that interact directly with the mPTP (i.e., chaperone networks), (iii) desensitization through kinase signaling pathways, and (iv) inhibition mediated by metabolic changes. All of these mechanisms do not necessarily occur in the same tumor type; however, they are not necessarily independent and can integrate with each other.

Interestingly, the establishment of this variety of integrated mPTP inhibition mechanisms may be the result of an evolutionary process. Opening of the mPTP may have acted as a selection pressure that only permitted the proliferation of cells capable of evading the MPT induction mechanism.

It may be possible to generate such selection pressure via autophagy, the process by which cytoplasmic constituents are sequestered into double-membrane vesicles called autophagosomes and selectively degraded by fusion of the autophagosomes with lysosomes. Opening of the mPTP can lead to loss of Ψm, with subsequent swelling of the mitochondrial matrix.

It has been reported that damaged and depolarized mitochondria are sequestered into autophagic vacuoles in a selective form of autophagy called “mitophagy” (132, 133). In 1997, Lemaster’s group initially proposed that the MPT could mediate the depolarization required for selective degradation of mitochondria through autophagy (134). These researchers observed spontaneous mitochondrial depolarization preceding organelle fusion with lysosomes (presumably through autophagosome and autophagolysosome formation), and the frequency of these events increased dramatically when autophagy was induced by serum starvation.

Strong support for this hypothesis was provided in a more recent study in which the authors showed that mitochondrial depolarization (induced by serum starvation) was dramatically inhibited by administration of the CypD inhibitor CsA. CsA administration, in addition to knocking down CypD, prevents mitochondrial sequestration into autophagosomes, indicating that serum starvation leads to mPTP opening that in turn drives selective mitochondrial degradation (here, the MPT was detected as mitochondrial depolarization, which, as previously stated, should be used carefully and in combination with the appropriate controls).

The connection between MPT and autophagy in cancer is less clear, mostly because knowledge regarding autophagy and cancer is scarce, and the data are controversial.

In fact, several autophagy genes have been found to be compromised in several types of cancers, and overexpression of some of these genes results in autophagy activation and growth inhibition (135–137). However, it has also been observed that nutritional stress (low availability of nutrients, oxygen, and mitogens) in the tumor environment leads to autophagy activation that in turn promotes tumor survival. Despite this finding, different studies have indicated that dysfunctional mitochondria accumulate in cancer, apparently due to dysfunctional mitophagy, which facilitates the accumulation of ROS (see above) and favors the metabolic reprograming that occurs in cancer (138–141).

Considering the previous findings regarding MPT and mitophagy, it could be speculated that an increase in the mPTP threshold occurs early in tumor transformation, impeding the spontaneous depolarization that should “mark” stressed mitochondria for selective degradation. This would lead to accumulation of mitochondria and a continuation of ROS generation, which in turn triggers the proliferative pathway for tumor formation.

Unfortunately, no studies have reported findings that are sufficient to validate this hypothesis; however, some studies on cancer stem cells (CSCs) have provided clear evidence. CSCs are cells that are able to undergo self-renewal and are committed to a cancer cell lineage, thus generating a tumor. The existence of these cells was initially hypothesized as part of a model that would explain the origins of tumor heterogeneity; however, their existence has finally been confirmed in several types of cancer (142–145), and they are now intensively being investigated. In a recent paper by Vega-Naredo and co-workers, several mitochondrial features were investigated during the differentiation of a cell line derived from a teratocarcinoma called P19 (146). This embryonic carcinoma cell line is pluripotent and can be induced in immortalized endodermal or mesodermal cells by retinoic acid. In this study, the authors observed that during the differentiation process, mPTPs switch from a more opened state to a more closed state. Although the effect of retinoic acid on mPTPs was not addressed, this study supports the possibility that cancer cells have evolved mechanisms to escape mPTP opening by desensitizing mPTPs.

In addition, several strategies designed to rescue mPTP activity have shown significant success [for extensive reviews, see in Ref. (74, 147, 148)].

Overall, a growing body of evidence suggests that inhibition of the mPTP may be a protective mechanism for cancer cell survival and proliferation and that targeting the mPTP may be a promising strategy for improving anticancer therapies.

## Conflict of Interest Statement

The authors declare that the research was conducted in the absence of any commercial or financial relationships that could be construed as a potential conflict of interest.

## Abbreviations

ANT, adenine nucleotide translocator; Bax, Bcl-2-associated protein X; Bak, Bcl-2 homologous antagonist killer; Bcl-2, B-cell lymphoma 2; Bcl-Xl, B-cell lymphoma-extra large; CypD, cyclophilin D; CK, creatine kinase; CsA, cyclosporine A; ER, endoplasmic reticulum; GSK3β, glycogen synthase kinase 3 beta; HIF1α, hypoxia-inducible factor 1 alpha; HKII, hexokinase 2; H-RAS, Harvey rat sarcoma viral oncogene homolog; HSP60, heat shock protein 60; HSP90, heat shock protein 90; IMM, inner mitochondrial membrane; IP3R, inositol phosphate 3 receptor; MAMs, mitochondria-associated membranes; Mcl-1, myeloid cell leukemia 1; MPT, mitochondria permeability transition; mPTP, mitochondrial permeability transition pore; OMM, outer mitochondrial membrane; PKC, protein kinase C; PML, promyelocytic leukemia; PP2A, protein phosphatase 2A; PTEN, phosphatase and tensin homolog; ROS, reactive oxygen species; SOD-2, superoxide dismutase-2; TRAP-1, TNF receptor-associated protein-1; TRX-2, thioredoxin reductase; TSPO, translocator protein; VDAC, voltage-dependent anion channel; WNT, wingless-type MMTV integration site family member.
